# Protection and pathology in TB: learning from the zebrafish model

**DOI:** 10.1007/s00281-015-0522-4

**Published:** 2015-09-01

**Authors:** Annemarie H. Meijer

**Affiliations:** Institute of Biology, Leiden University, Einsteinweg 55, 2333 CC Leiden, The Netherlands

**Keywords:** *Mycobacterium marinum*, Tuberculosis, Granuloma, Innate immunity, Inflammation, Autophagy

## Abstract

Zebrafish has earned its place among animal models of tuberculosis. Its natural pathogen, *Mycobacterium marinum*, shares major virulence factors with the human pathogen *Mycobacterium tuberculosis*. In adult zebrafish, which possess recombination-activated adaptive immunity, it can cause acute infection or a chronic progressive disease with containment of mycobacteria in well-structured, caseating granulomas. In addition, a low-dose model that closely mimics human latent infection has recently been developed. These models are used alongside infection of optically transparent zebrafish embryos and larvae that rely on innate immunity and permit non-invasive visualization of the early stages of developing granulomas that are inaccessible in other animal models. By microinjecting mycobacteria intravenously or into different tissues, systemic and localized infections can be induced, each useful for studying particular aspects of early pathogenesis, such as phagocyte recruitment, granuloma expansion and maintenance, vascularization of granulomas, and the phagocyte-mediated dissemination of mycobacteria. This has contributed to new insights into the mycobacteria-driven mechanisms that promote granuloma formation, the double-edged role of inflammation, the mechanisms of macrophage cell death that favor disease progression, and the host-protective role of autophagy. As a result, zebrafish models are now increasingly used to explore strategies for adjunctive therapy of tuberculosis with host-directed drugs.

## Introduction

*Mycobacterium tuberculosis* (*Mtb*) is one of the most successful human pathogens that is estimated to have infected one third of the human population and to be responsible for nine million new cases of tuberculosis (TB) in 2013 (WHO Global Tuberculosis report 2014). *Mtb* parasitizes macrophages and can persist for decades as a latent infection inside its human host [1]. The formation of granulomas is central to the pathology of TB and the development of latency [2, 3]. TB granulomas are highly organized host cellular structures that contain an inner core of infected macrophages and necrotic cell debris (the caseum) where bacteria persist extracellular. In the surrounding cell layers, other immune cells, including dendritic cells, neutrophils, and T and B cells, wall off the bacteria inside the granuloma [2, 4]. A latent infection in granulomas has the ability to reactivate after many years, and the disease can be transmitted when granuloma integrity is lost. An alarming rise in antibiotic resistances and the lack of an effective vaccine against latent or reactivated TB emphasize the need for novel therapeutic strategies to control TB [5].

Animal models are indispensable for studying the host and bacterial factors involved in TB pathology and for evaluating new drug and vaccine candidates. Important insights into human TB pathology have been inferred from experimental *Mtb* infections in mice, guinea pigs, rabbits, and non-human primates, particularly macaques [6, 7]. In addition, now for over 10 years, the zebrafish has become widely used as an alternative animal model for TB [8–10]. Zebrafish can be infected with *Mycobacterium marinum* (*Mm*), a natural pathogen of cold-blooded vertebrates. The genomes of *Mtb* and *Mm* share 3000 orthologs with an average amino acid identity of 85 % [11]. *Mm* occasionally causes a granulomatous skin infection in humans known as “fish tank granuloma” [12]. In zebrafish, *Mm* causes a systemic disease with containment of bacteria in granulomas that show strong structural similarity with the human TB granuloma [13–15]. Although differences in the adaptation of *Mtb* and *Mm* to different hosts must not be ignored, the important virulence factors of *Mtb* are functionally able to complement mutations in *Mm* genes and vice versa [16, 17].

Studies using the zebrafish-*Mm* model have contributed importantly to the changed view of the role of the granuloma in TB pathogenesis that has emerged over the recent years [2, 10]. Historically, the granuloma has been regarded as a static host defense structure. However, granuloma formation is driven by bacterial virulence, and it is now widely accepted that granulomas are highly dynamic structures that, especially during early stages of pathogenesis, can promote the dissemination of mycobacteria [2, 18]. Work in zebrafish has shown that the presence of macrophages is sufficient to initiate granuloma formation [19]. This means that the early stages of granuloma formation can be observed in optically transparent zebrafish embryos and larvae that have a functional innate immune system but have not yet developed adaptive immunity. The use of these zebrafish early life stages has shown that secondary granulomas can be seeded by the egression of infected macrophages from a primary granuloma [20]. That granulomas are not impenetrable is evidenced by experiments with superinfecting mycobacteria that are found to be transported by infected macrophages into established granulomas. This was initially observed during *Mm* infection of zebrafish and frogs and has subsequently been confirmed during *Mtb* infection in mice [21, 22]. Intravital imaging in both zebrafish and mice has demonstrated the migration of immune cells throughout the process of granuloma development [20, 23]. The heterogeneity and dynamic nature of granulomas observed in zebrafish and mice is in perfect agreement with serial PET-CT imaging data from *Mtb*-infected cynomolgus macaques showing that individual granulomas within the same host can regress and even be sterilized, while other granulomas progress during the same time [24]. This review will discuss how studies either in adult zebrafish or in embryos and larvae have advanced our understanding of mycobacterial virulence factors and of host genes implicated in immune protection or TB pathogenesis.

## TB in adult zebrafish

While entry via de gastrointestinal tract is most likely the primary route of *Mm* infection in the natural environment, experimental infection of adult zebrafish is commonly achieved by intraperitoneal injection [13–15, 25]. Dependent on the particular dose and strain, the infection manifests with acute symptoms or develops as a chronic progressive disease [13–15]. Acute disease is characterized by rapid lethal inflammation and is more frequently observed with human-derived isolates of *Mm* that form a distinct genetic cluster [14]. Swelling of the abdomen, hemorrhages, and skin ulcerations are typically observed at the end stage of the chronic progressive disease [14]. This is associated with a strong induction of immune response genes and inflammation markers at the transcriptional level [26–28]. Well before external symptoms become apparent, well-organized granulomas are formed in different organs, including the liver, pancreas, kidney, intestines, and spleen and sometimes also in the connective tissues [13–15, 29]. Some intraperitoneally infected zebrafish also develop granulomas in close relation with brain tissue and meninges; therefore, the model can also be used to study TB meningitis [30].

Granulomas in adult zebrafish consist of tightly packed epithelial cells surrounding a central region where macrophages are the predominant cell type and mycobacteria are detectable by acid-fast staining [14]. Importantly, most granulomas in zebrafish show necrosis in the central core, and many are hypoxic [14, 15, 31]. Central necrosis is a hallmark feature of the human TB granuloma that has been difficult to reproduce in mouse and can be mimicked only in some newer mouse models of TB [32–34]. In addition, mature granulomas in zebrafish often are multi-centric and surrounded by a fibrous capsule [35]. Calcification of granulomas has not been observed in zebrafish, and their granulomas contain much less lymphocytes than those of human TB patients [15, 30]. Despite this lower number of lymphocytes, the function of adaptive immunity is critical for controlling TB in zebrafish since mutants in *rag1* are hypersusceptible to *Mm* infection [15]. The chronic progressive zebrafish infection model has proven useful for antimycobacterial drug screening as well as for testing of host-targeted drugs [31, 36]. Reducing vascularization of zebrafish granulomas by pharmacological inhibition of vascular endothelial growth factor (Vegf) signaling decreases overall infection burden, and fish display an increased frequency of sterilized granulomas [31]. Together with a study of *Mtb*-infected rabbits, this suggests the potential use of antiangiogenic drugs in combination with anti-TB drugs for treatment of TB patients [37].

The study of mechanisms underlying latency and reactivation of TB is hampered by the limitations of animal models. Recently, it has been shown that the zebrafish-*Mm* model can be used to mimic aspects of latent disease [35]. Several weeks after intraperitoneal injection with a low-dose of *Mm* bacteria, zebrafish developed stable bacterial loads and constant numbers of granulomas. Ex vivo activation by resuscitation promoting factor demonstrated the dormancy of Mm under these conditions. The development of latency relies on *rag1*-mediated adaptive immunity, and immunosuppression induced by gamma irradiation leads to reactivation of the dormant bacterial population. This model has much potential for preclinical testing of new drug and vaccine candidates. As a proof-of-principle, BCG vaccination and DNA vaccination with different mycobacterial antigens were shown to protect zebrafish from *Mm* infection in this model [38]. Zebrafish are not easily inbred, and therefore, large variations are often observed in studies using this model [39, 40]. However, the natural heterogeneity of the zebrafish population has been taken advantage of to gain understanding of genetic differences that are associated with the ability of individuals to control latent infection or that pose risk factors for reactivation [40]. This study showed that zebrafish individuals with well-controlled infection display not only an efficient Th1 immune response but also an adequate Th2 response. Zebrafish heterozygous for a mutation in *furinA*, encoding a proprotein convertase of Th1 cells, showed reduced mycobacterial load in the latency model, suggesting proprotein convertase inhibitors as potential drugs for TB [41].

A number of mutants in mycobacterial virulence genes have been tested in adult zebrafish [42–47]. *Mtb* and *Mm* use type VII secretion systems, named ESX-1 to ESX-5, to secrete proteins across their lipid-rich cell wall [48]. The ESX-1 system is a major virulence factor and absent in attenuated strains that carry the so-called RD1 deletion (ΔRD1), including the live vaccine strain *Mycobacterium bovis* BCG [49]. In zebrafish, several ΔRD1 *Mm* strains showed decreased virulence, supporting the usefulness of the fish model for TB [15, 42]. Infection with ΔRD1 *Mm* has been shown to delay the kinetics of granuloma formation, resulting in solitary and loose macrophage aggregates and very few necrotizing granulomas [15]. Similarly, as discussed further below, ΔRD1 delays the kinetics of granuloma formation in zebrafish embryos that only possess an innate immune system. However, in the case of an ESX-5 mutant, marked differences were observed between infections in zebrafish embryos and adults [29]. The ESX-5 secretion system is required for transport of proteins of the PE and PPE families, of which the functions remain largely unknown [48]. While ESX-5-deficient *Mm* is slightly attenuated in zebrafish embryos, it turned out to be more virulent in adults, causing rapid development of necrotizing granulomas accompanied by increased expression of proinflammatory genes [29]. The different response of embryos and adults to ESX-5 mutants is likely not mediated by the adaptive immune system, since ESX-5 mutants still have a growth advantage over wild-type *Mm* in *rag1*-deficient zebrafish. This study indicates that *Mm* relies on ESX-5-mediated protein secretion for establishing persistent infection and highlights that parallel use of embryo and adult zebrafish models can be important for unraveling mycobacterial virulence mechanisms [29].

## TB in zebrafish embryos and larvae

**Fig. 1:**
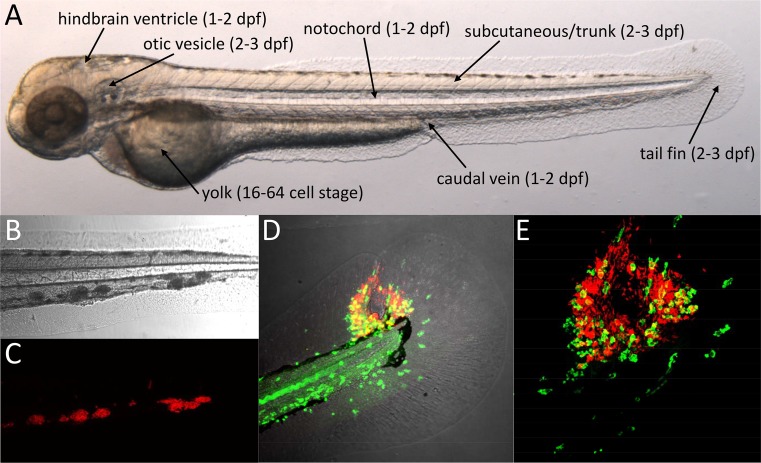
*Mm* infection of zebrafish embryos. **a** Two-day-old zebrafish embryo showing the different sites used for microinjection of *Mm*. The developmental stages at which these injections are usually performed are indicated between *brackets*. The location of trunk injection is similar to that of subcutaneous injection but the microinjection needle is inserted deeper into the tissue. **b**–**c** Confocal transmission (**b**) and fluorescence (**c**) images showing a detail of the tail of a 5-day-old larvae with red fluorescent *Mm* in granuloma-like aggregates at 4 dpi into the caudal vein. **d**–**e** Granuloma with central necrosis in the tail fin of a zebrafish larva at 5 dpi. Leukocytes detected by L-plastin antibody staining are shown in *green* and *Mm* in *red*. Images show an overview of the tail fin infection (**d** confocal transmission and fluorescence overlay) and a detail of the granuloma (**e** confocal fluorescence)

The external fertilization of zebrafish eggs provides easy access to developing embryos. Embryos naturally hatch by 2 days post fertilization (dpf), but the chorion can be removed at 1 dpf to facilitate experimental infection. By 72 h post fertilization (hpf), embryos reach the larval stage and larvae become capable of independent feeding by 5 dpf [50]. During this developmental time period, the primary functional immune cell types are the macrophages and neutrophils; thus, immunity relies on the innate arm of the system [51–53]. Embryos and larvae develop normally under anesthesia with tricaine methane sulfonate (MS222), which makes them ideal for non-invasive time lapse imaging. A growing collection of fluorescent reporter lines facilitates the visualization of different immune cell types, subcellular structures, and the activation of immune response genes [54]. Knockdown studies in zebrafish embryos using antisense morpholino oligonucleotides have strongly contributed to the understanding of early mycobacterial pathogenesis [55–57]. In addition, random mutagenesis screens proved a useful source of zebrafish mutants for TB research [39, 58, 59]. Recent advances in gene targeting technology should lead to an increased use of knockout lines in both embryo/larval and adult TB models [60, 61]. This will also enable gene disruption in a cell- or tissue-specific manner, which until now has been a limitation of the zebrafish model [62]. Zebrafish embryos and larvae are particularly useful for screens of anti-TB drugs that can be added simply to the medium [63, 64]. However, not all compounds are efficiently taken up via the skin, and therefore, drug efficacy in this system should be correlated with uptake characteristics in order to eliminate false negatives and to enable better comparison with tests in mammalian models [65]. Systemic or localized infection of embryos and larvae can be achieved by microinjecting *Mm* bacteria at different sites, each of which has specific advantages to address different research questions (Fig. 1a) [66, 67].

### Intravenous infection

Systemic infection via the intravenous route was used in the first description of the embryo TB model and has since been the most frequently used [19]. The earliest opportunity for intravenous infection is shortly after the onset of blood circulation at 26 hpf, but microinjection into the vascular system can also be performed at later stages [66, 67]. *Mm* bacteria delivered into the blood are predominantly phagocytosed by macrophages [19, 68]. This is a rapid process, with a dose of around 200 CFU being internalized within 30–60 min [69]. At 3 days post infection (dpi), infected and non-infected macrophages are visible in tight granuloma-like aggregates spread over the larval tissues but mostly occurring in the proximity of blood vessels in the ventral part of the tail, in the area of a temporary hematopoietic site, named the caudal hematopoietic tissue (Fig. 1b, c) [19, 70, 71]. Most intravenously infected embryos also develop aggregates in different areas of the brain, and this still occurs when bacteria are injected at a later time point (4 dpf) when the blood brain barrier has been formed [30]. Electron microscopy has shown that some of the infected macrophages in larval granuloma-like aggregates display an epithelioid morphology and that multi-nucleate giant cells are present, which are distinctive features of mature granulomas [19]. Furthermore, since *Mm* bacteria in these aggregates express granuloma-specific fluorescent reporter genes, the microenvironment appears to be similar to that in mature granulomas [19, 70]. The environment of these larval granulomas has been shown to favor the rapid development and dissemination of a multi-drug-tolerant intracellular *Mm* population [72]. Bacterial efflux pump inhibitors like verapamil can reduce this tolerance, demonstrating that the system can be used for investigating the mechanisms underlying tolerance and for therapeutic approaches to overcome tolerance [72, 73]. Infection with labeled *Mm* strains allows easy assessment of granuloma numbers, individual granuloma sizes, and overall bacterial burden from fluorescence images, making the intravenous infection model well suited to analyze the function of bacterial virulence factors and host genes as well as for evaluating drug effects on granuloma formation [57, 58, 64, 70, 74].

### Yolk infection

Injection of *Mm* into the yolk of developing embryos between the 16–128 cell stage leads to an infection that initially remains restricted to this area where macrophages do not enter but from 3 dpi spreads by an unknown mechanism into the larval tissues [75]. Once the infection spreads, bacteria are taken up by macrophages and granulomas form similar to those in the intravenous infection model. The yolk injection method finds use in drug screening as it can be automated using a robotic injector [63, 65]. Using the yolk as injection site, it has been shown that not only *Mm* but also *Mtb* bacteria can spread into larval tissues and survive inside macrophages [63]. Zebrafish are normally maintained at 28 °C, but the temperature was increased to 34 °C to support growth of *Mtb*. However, due to the slower growth of *Mtb*, the formation of granuloma-like aggregates by infected macrophages has not been observed in zebrafish larvae.

### Hindbrain ventricle infection

The hindbrain ventricle (fourth ventricle of the brain) is a cavity filled with cerebrospinal fluid into which macrophages can be recruited after injection of bacteria or chemotactic proteins and lipids [51, 59, 76]. This is a convenient injection site to study host and bacterial factors involved in chemotaxis or contributing to dissemination of *Mm* [59, 76]. While a large bacterial cluster develops locally in the hindbrain, macrophages can exit the ventricle and carry *Mm* to distal locations in the head, trunk, or tail regions [59, 76, 77]. Hindbrain injection has also been used to compare the ability of different strains to establish infection in embryos that received low-dose inocula of 1–3 *Mm* bacteria, probably similar to the natural infection dose of *Mtb* in human infections [76].

### Otic vesicle infection

Injection of bacteria into the cavity of the developing ear is an alternative possibility to create a localized infection that is useful for studying leukocyte recruitment and mobilization of macrophages and neutrophils at distal locations [59, 78].

### Notochord infection

The notochord consists of a longitudinal column of vacuolated cells surrounded by a sheath of collagen and serves as an embryonic skeleton prior to the formation of bone. This structure is inaccessible to macrophages and neutrophils and hypersusceptible to *Mm* infection [79, 80]. The virulence of an *Mm* TesA mutant defective for major cell wall lipids is retained in the notochord, while this mutant is strongly attenuated when injected intravenously [79]. Since macrophages and neutrophils accumulate in the periphery of an infected notochord, this model is useful for studying the host inflammatory response [80]. It has also been suggested as a model for the initial events characterizing bone tuberculosis [79].

### Subcutaneous infection

When *Mm* is injected into fluid-filled compartments, such as the blood or hindbrain ventricle, phagocytosis is dominated by macrophages [68, 77]. However, neutrophils play a major role in phagocytosis of mycobacteria in other models as well as in human TB infection [81, 82]. Neutrophils require a surface for efficient phagocytosis and have been shown to take up *Mm* bacteria when injected subcutaneously into zebrafish larvae [83, 84]. Therefore, subcutaneous injection or injection into other tissues such as muscle or the tail fin (described below) is useful to address the contribution of neutrophils to the early stages of mycobacterial pathogenesis [84–86].

### Tail fin infection

The larval tail fin consists of two epithelial cell layers on both sides with mesenchymal cells, extracellular matrix, and collagenous fibers in between. Injection of *Mm* into the tail fin results in the rapid attraction of macrophages and neutrophils and formation of a single granulomatous lesion [86]. This method enables visualizing the process of granuloma development from the first infected cell to the stage where a necrotic center is formed. The necrotic center is eventually extruded from the thin tissue of the tail fin, resulting in central pore (Fig. 1d, e) [86]. This thin tissue is very suitable for high-resolution microscopic imaging and has been used for correlative fluorescence and electron microscopy analysis of the formation of autophagic vesicles during *Mm* infection [86].

### Trunk infection

Injection of *Mm* into the dorsal region of the trunk leads to the formation of primary granulomas that grow larger than granulomas in other more vascularized areas and that develop local hypoxia. The trunk is therefore a preferred injection site for studying the association between granuloma formation and angiogenesis [31]. Trunk granulomas attract new vessels sprouting from the existing intersegmental vessels, and this response can be inhibited either by genetic depletion of macrophages or by pharmacological inhibition of the Vegf receptor. Blockade of Vegf signaling also reduces vascular leakiness, dissemination of *Mm*, and overall bacterial burden. Production of Vegf is independent of hypoxia development, and macrophages on the edges of the developing granulomas are proposed to be the source of this proangiogenic signaling molecule [31].

## Roles of macrophages and neutrophils during early pathogenesis

**Fig. 2:**
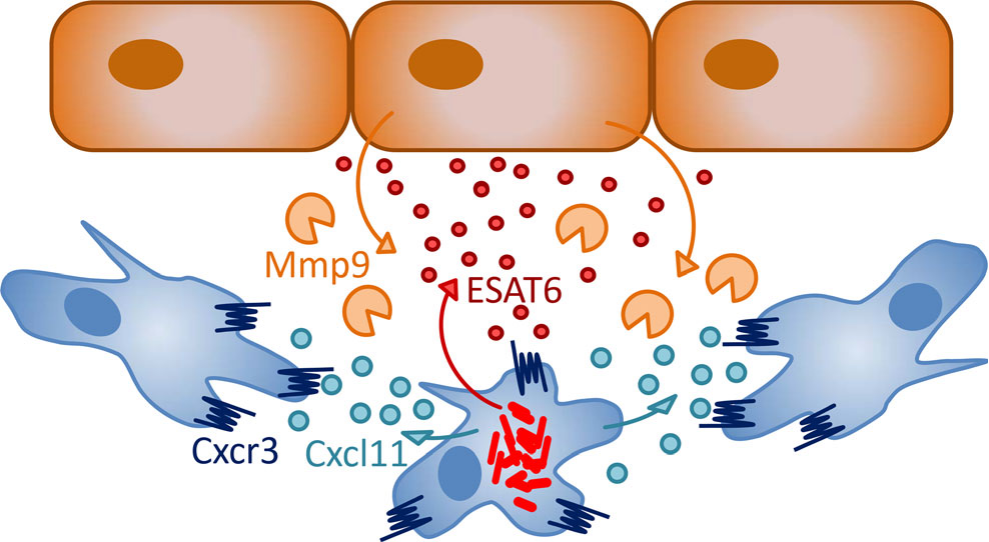
Signals involved in early granuloma formation. Studies in zebrafish suggest that mycobacteria (*red*) inside infected macrophages (*blue*) secrete the ESAT6 virulence factor, which, in turn, induces nearby epithelial cells (*brown*) to secrete the matrix metalloproteinase Mmp9 that is thought to facilitate the migration of macrophages [55]. This matrix degradation pathway could act cooperatively with Cxcr3-Cxcl11 signaling between infected and uninfected macrophages promoting the chemoattraction of macrophages and their aggregation into initial granulomas [59]

Genetic depletion of macrophages during embryo development showed that macrophages limit the growth of *Mm*, yet are essential for the dissemination of *Mm* into tissues [77]. Macrophages phagocytose *Mm* in a manner partially dependent on the conserved scavenger receptor Marco [69]. Several factors have be shown to be involved in the containment of infection by macrophages in zebrafish, including tumor necrosis factor (Tnf), autophagy components (P62/Sqstm1 and Dram1), and a macrophage-specific perforin (Mpeg1) [57, 87, 88]. In contrast, *Mm* bacteria seem to be able to evade reactive oxygen or nitrogen-mediated defenses [56, 76, 89]. *Mm* bacteria initially replicate inside membrane-enclosed compartments of macrophages from which they eventually escape in a manner requiring the ESX-1 secretion system [57, 86]. By a mechanism also requiring ESX-1, uninfected macrophages in the vicinity of infected cells polarize, increase their motility, and scavenge dying infected cells, thereby driving expansion of the granuloma [20]. The ESX-1-secreted factor ESAT6 is thought to act as a signal that induces epithelial cells to secrete the matrix metalloproteinase Mmp9, facilitating the recruitment of macrophages and expansion of granulomas (Fig. 2) [55]. Rifampicin-loaded nanoparticles are rapidly taken up by macrophages and reduce bacterial load of zebrafish larvae, showing that the property of macrophages to be recruited to granulomas can be exploited for drug delivery [71]. It remains to be elucidated how macrophages can reverse migrate and egress from granulomas to disseminate *Mm*, and future work in zebrafish larvae may help to answer this question [20, 54, 59].

Zebrafish embryo/larval models are also helping to clarify the role of neutrophils during early mycobacterial pathogenesis. Bacteremia preceding the death of larvae at later stages of *Mm* infection is associated with neutropenia, suggesting that depletion of neutrophils affects the ability to control infection [84]. In agreement, increased bacterial burden is observed in a zebrafish transgenic line ectopically expressing a gain-of-function truncation of chemokine receptor Cxcr4 that causes retention of neutrophils in the hematopoietic tissues [68, 90]. In wild-type larvae, neutrophils attracted to granulomas around 3 dpi have been observed to phagocytose dying infected macrophages. A subset of these neutrophils is able to kill intracellular *Mm* through NADPH oxidase-mediated reactive oxygen production [68]. At earlier stages of infection, when *Mm* is mostly restricted to macrophages, uninfected neutrophils respond by production of nitric oxide, detected by increased levels of nitrotyrosine [91]. However, this response is not an effective defense mechanism, since blocking of inducible nitric oxide synthase (iNos/Nos2a) has no effect on the ability to control *Mm* infection. In contrast, host defense is enhanced when the nitric oxide response of neutrophils is artificially upregulated prior to infection, not allowing the bacteria time to adapt. This can be achieved by genetic or pharmacologic manipulation of hypoxia-inducible factor (Hif-α) signaling, suggesting this pathway as a potential host-therapeutic target [89, 91]. That neutrophils contribute to early host defense against mycobacteria is supported by studies in other animal models, but there is also much evidence for a pathological role of neutrophils in driving inflammation and progression of TB disease [82, 92–94].

## Mycobacterial avoidance and exploitation of Toll-like and chemokine receptor responses

Zebrafish embryos carrying a mutation in Myd88, the common adaptor of Toll-like and interleukin-1/18 receptors, show increased susceptibility to *Mm* infection following intravenous injection [39, 76]. In this systemic infection model, Myd88 deficiency has been shown to impact on multiple pathways of innate host defense against *Mm*, including cytokine-mediated, nitrosative, and autophagic defense mechanisms [39, 57, 89]. In contrast, it has been found that Myd88 is not required for the initial recruitment of macrophages in a local hindbrain infection model, indicating that mycobacteria have evolved mechanisms to avoid TLR/Myd88-mediated defenses [76]. Mycobacterial cell wall lipids have been implicated in this immune evasion strategy, notably the phthiocerol dimycocerosates (PDIM) known to be major virulence factors of pathogenic mycobacteria. PDIM-deficient *Mm* strains or other bacterial species not containing PDIM (*Mycobacterium smegmatis*, *Staphylococcus aureus*, *Pseudomonas aeruginosa*) trigger a TLR/Myd88-dependent response. Zebrafish macrophages recruited to these strains show a microbicidal iNos-positive phenotype, while macrophages recruited to PDIM-expressing *Mm* are iNos negative. In agreement, PDIM-deficient *Mtb* attract a higher number of iNos-positive cells in lung tissue of mice compared with H37Rv *Mtb* [76]. These findings led to an interesting model proposing that pathogenic mycobacteria use PDIM to mask the underlying TLR ligands and thereby establish infection in a permissive macrophage population that is encountered in the lower respiratory tract where *Mtb* is known to initiate infection rather than in the upper tract where TLR/Myd88-dependent macrophage polarization is induced by the presence of resident microflora and inhaled environmental microbes [76, 95]. Besides this role in masking TLR recognition, PDIM lipids are likely to impact directly on the microbicidal activity of macrophages through their capacity to insert into the plasma membrane and into the membranes of intracellular compartments where mycobacteria replicate [96].

The CCL2-CCR2 chemokine signaling axis has been linked to the recruitment of permissive macrophages by PDIM-containing *Mm*, in a manner dependent on phenolic glycolipids (PGLs) [76]. CCR2 is required for the mobilization of monocytes from the bone marrow and their trafficking to sites of inflammation [97]. Consequently, in murine models of infectious diseases, including TB, CCR2 deficiency impairs host defense [98–100]. However, as pointed out by Cambier et al., experimental infections using high inocula may have failed to reveal how mycobacteria can exploit CCL2-CCR2 signaling to establish infection under clinically relevant low inoculum conditions [76]. While CCL2 is generally considered an inflammatory chemokine, there is evidence that it can shift the polarization of macrophages toward an antiinflammatory phenotype [101]. This is consistent with the model proposing that CCL2-CCR2 signaling promotes mycobacterial infectivity under low inoculum conditions and with a genetic association study correlating high expression of CCL2 with TB susceptibility [76, 102].

Mutation of *cxcr3.2*, one of three zebrafish homologs of the human CXCR3 receptor, has a similar effect on macrophage recruitment to *Mm* infection in the zebrafish hindbrain injection model as knockdown of Ccl2-Ccr2 signaling [59]. It is currently not known if these two chemokine-mediated recruitment mechanisms act redundantly or in a concerted manner. The interferon-γ-inducible inflammatory chemokines CXCL9, 10, and 11, the ligands of human CXCR3, show enhanced plasma levels in TB patients, and CXCR3 ligands are also expressed in pulmonary granulomas of *Mtb*-infected cynomolgus macaques [103, 104]. Besides reducing macrophage recruitment in response to locally injected *Mm* bacteria or Cxcl11-like chemokines, *cxcr3.2* mutation also results in other phenotypes suggesting that mycobacteria use CXCR3 signaling to their advantage. First, *cxcr3.2* mutation reduces dissemination of *Mm* from the hindbrain ventricle to other regions of the head, trunk, and tail. Second, *cxcr3.2* mutation reduces the expansion of granulomas, either those resulting from disseminated local infection or those resulting from systemic intravenous infection. Third, *cxcr3.2*-deficient macrophages have reduced basal motility. Although this motility defect can be overcome by delivering Cxcr3.2-independent stimuli, it might limit spreading of mycobacteria between macrophages in granulomas (Fig. 2) [59]. A host-beneficial effect of disrupting the CXCR3 axis is not limited to the context of macrophage function in zebrafish larvae. *CXCR3*-deficient mice control chronic *Mtb* infection better than wild-type animals, and this has been attributed to an adverse effect on T cell priming [105]. In another study, it has been shown that *CXCR3*-deficient mice are delayed in granuloma formation similar to neutrophil-depleted mice in which the expression of CXCR3 signaling chemokines is diminished [106]. It has recently been suggested that CXCR3 deficiency in mice is linked with polarization of macrophages toward an iNos-negative, antiinflammatory phenotype [107, 108]. If such polarization would occur in the context of mycobacterial infection, this would make macrophages more permissive for bacterial growth, which is contrast with the host-beneficial effect of CXCR3 deficiency in both zebrafish and murine TB models. Together, these studies indicate that, rather than iNos-mediated defense, other CXCR3-dependent mechanisms are important for control of mycobacterial infection and support further investigation of the CXCR3-CXCL11 axis as a host therapeutic target for TB treatment [59, 105, 106].

It currently remains unanswered if there are pre-existing macrophage subsets in zebrafish embryos responding to Ccl2- or Cxcl11-like chemokines and Myd88-dependent cues, or if these signals might drive different polarization of recruited macrophages. The source of the chemoattractants also remains to be established. Two not mutually exclusive origins of infection-inducible chemokines are the neuroepithelial cells lining the hindbrain ventricle or the macrophages themselves. Few macrophages can be resident in the cavity prior to injection or are initially attracted independent of the bacterial presence due to a minor wounding effect that is unavoidable in this assay. In situ mRNA detection of the chemokines and their receptors is unfortunately limited by low expression levels. However, RNAseq of leukocyte populations isolated by fluorescent-activated cell sorting suggests that macrophages could indeed be the source of both CCL and CXCL chemokines [109 and unpublished results].

## Protective and pathological roles of inflammation

The dual role that inflammation plays in TB pathogenesis is extensively discussed in recent reviews [10, 110, 111]. Consistent with many studies in other animal models, zebrafish larvae are found to be hypersusceptible to *Mm* infection either when inflammation fails or when the inflammatory response is exacerbated [39, 56, 58, 87, 112–114]. The optical transparency of zebrafish larvae has helped to distinguish whether defects in inflammation affect the early formation of granulomas or their maintenance. Knockdown of the Tnf receptor in zebrafish accelerates granuloma formation but leads to rapid breakdown of granulomas and extracellular growth of *Mm* [87]. Limited Tnf production leads to the same phenotype, supporting that Tnf is dispensable for granuloma formation but critical for the maintenance of granuloma integrity [58]. Increased expansion of *Mm* granulomas in Myd88-deficient zebrafish larvae agrees with these findings [39, 76]. However, it is likely that multiple factors contribute to this phenotype, since lack of Myd88-dependent signaling reduces not only *tnf* gene expression but also the expression of other major cytokine and defense genes [39, 57, 88].

A zebrafish mutagenesis screen uncovered an intricate cross talk between cytokine and lipid mediators of inflammation during *Mm* infection [58]. In hypersusceptible *lta4h* mutants, deficiency in leukotriene A4 hydrolase redirects eicosanoid intermediates into the production of antiinflammatory lipoxins, which, in turn, limits Tnf production [58]. Other intersections between the cytokine and eicosanoid networks have recently been revealed, notably the production of prostaglandin E2 (PGE2) driven by IL-1, which promotes the control of *Mtb* infection [115]. It is now widely believed that a better understanding of the complexity of this interplay holds promise for immunotherapeutic interventions using clinically approved drugs to carefully manipulate the cytokine/eicosanoid balance in TB patients [110, 111, 115].

A hyperinflamed status can be induced in zebrafish larvae by injection of recombinant TNF, by overexpression of the leukotriene biosynthetic enzyme Lta4h, or by knockdown of the non-receptor tyrosine phosphatase Ptpn6, a negative regulator of inflammation [56, 88, 112, 113]. In all cases, this results in hypersusceptibility to *Mm* infection, underscoring the importance of a balanced inflammatory response. Mechanistically, the detrimental effect of high levels of Tnf in zebrafish has been attributed to the mode of cell death of *Mm*-infected macrophages in this situation [56]. Excess Tnf triggers the production of mitochondrial reactive oxygen species through Rip1-Rip3-dependent signaling, and this induces a programmed type of necrotic cell death. This necroptotic cell death is mediated by cyclophilin D, which is involved in formation of the mitochondrial permeability transition pore complex and by the lysosomal acid sphingomyelinase, which is required for ceramide production. Pharmacological inhibition of these two pathways prevents initiation of the necroptotic program and reverses the hypersusceptibility of zebrafish larvae with high Tnf levels [56]. In the case of low Tnf levels, macrophages likely undergo passive necrosis with a similar exacerbating effect on *Mm* infection as the induction of the necroptotic pathway [87]. These results are in line with evidence that virulence factors of *Mtb* trigger necrotic cell death of macrophages, while inhibiting the immunologically silent apoptotic cell death program [116]. Besides TNF, lipid mediators are crucial for the mode of cell death, with necrosis being promoted by antiinflammatory lipoxins and inhibited by PGE2 [117]. These recent insights in the impact of the cell death program on the outcome of mycobacterial infection have important therapeutic implications [116]. Zebrafish larvae are a useful model to test cell death modulators in vivo, since mechanisms of cell death appear to be strongly evolutionary conserved [56].

## Protective role of autophagy

**Fig. 3:**
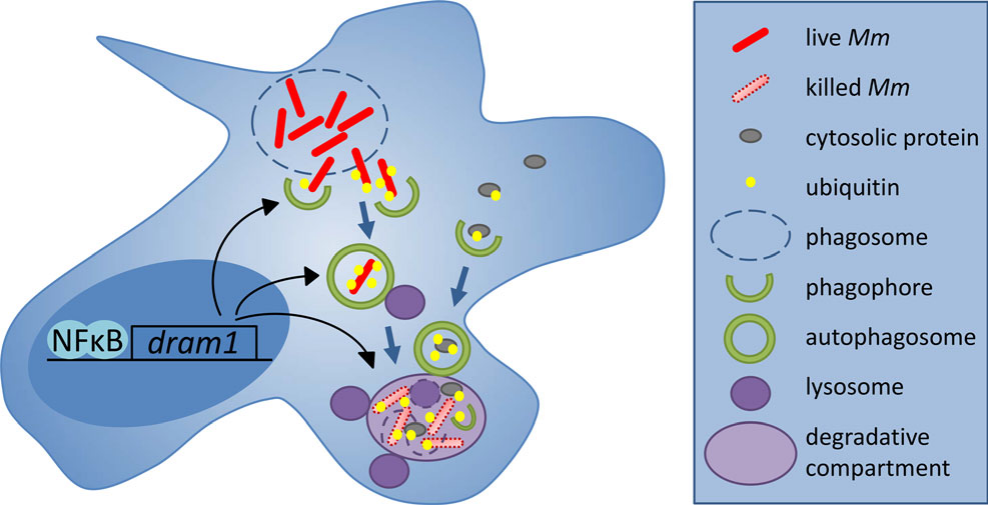
Dram1-modulated autophagic defense pathway in macrophages. Following infection of zebrafish embryos, *Mm* bacteria are detected inside membrane compartments of macrophages as well as freely in the cytoplasm [57, 86]. Translocation of *Mm* to the cytoplasm is dependent on the ESX-1 secretion system required for rupture of the phagosome membrane (*dashed line*). By analogy with studies of *Mtb* in cultured macrophages, *Mm* bacteria escaping the phagosome are thought to be ubiquitinated by a STING-dependent pathway and targeted to selective autophagy mediated by ubiquitin receptors [57, 124]. DRAM1 is induced during infection by Myd88-NFκB signaling and proposed to promote the formation of autophagosomes as well as multiple vesicle fusion events between autophagosomes and lysosomes leading to the formation of larger degradative compartments [57, 125]. The microbicidal properties of these compartments could be enhanced due to the delivery of ubiquitinated peptides by autophagosomes [126]

The recognition of autophagy as an innate host defense mechanism against intracellular pathogens started with the observation that stimulation of autophagy by nutrient starvation or rapamycin treatment could overcome the *Mtb*-induced block in phagolysosome maturation [118, 119]. Since then, a number of siRNA and chemical screens in Mtb- or BCG-infected cells have pointed toward autophagy as a therapeutic target for TB treatment [120–123]. During autophagy (or macroautophagy), protein aggregates, organelles or intracellular bacteria become enclosed in autophagosomes characterized by a double membrane and the marker protein Lc3. This can be a non-specific bulk process or a selective process mediated by specific cargo receptors, such as the ubiquitin receptors p62 (sequestosome 1), optineurin, and ndp52 [119]. Selective autophagy by the receptor-mediated pathway requires that mycobacteria escape from the phagosomal compartment or induce damage to the phagosomal membrane permitting them to be ubiquitinated (Fig. 3). A functional ESX-1 secretion system is required for the rupture of phagosomes and ubiquitination of mycobacteria [124, 127, 128]. However, ESX-1-deficient BCG bacteria are also sensitive to autophagy stimulation, indicating that mycobacteria can be targeted to autophagy via multiple mechanisms that may include sequestering of complete phagosomes by autophagosomal isolation membranes, the recruitment of Lc3 to phagosomes (Lc3-associated phagocytosis), or the formation of amphisomes through the fusion between autophagosomes and endosomes [118, 129, 130]. In zebrafish larvae, the escape-dependent autophagy route appears to predominate and ESX-1-deficient *Mm* fail to recruit Lc3 [57, 86]. Confocal imaging of GFP-Lc3 transgenic zebrafish combined with electron microscopy has confirmed the presence of wild-type *Mm* in compartments with autophagic morphology [86]. Approximately two thirds of GFP-Lc3-positive *Mm*-containing vesicles in leukocytes of larval tail fin granulomas stain positive for a lysosomal marker [86]. Furthermore, imaging in zebrafish has revealed the frequent presence of small GFP-Lc3 vesicles in close vicinity of bacteria or bacterial aggregates [57, 86]. These vesicles might serve to deliver neo-antimicrobial peptides to the *Mm*-containing compartments, a process that has been shown to augment the bactericidal properties of autophagic organelles in Mtb-infected cells (Fig. 3) [126].

Rapamycin induces autophagy via mTOR kinase but also leads to immunosuppression [131]. While promoting intracellular killing of *Mtb* in vitro, rapamycin is detrimental to zebrafish host defense against *Mm* [57, 118]. Interestingly, a recent screen for mTOR-independent inducers of autophagy has shown that a clinically approved anticonvulsant drug, carbamazepine, triggers autophagy by a novel myo-inositol dependent pathway and is effective in vivo, both against *Mtb* in mice and against *Mm* in zebrafish [122]. Another potential therapeutic target is the DRAM1-mediated autophagy pathway that we have recently found to protect against *Mm* infection in zebrafish (Fig. 3) [57, 125]. DRAM1 is a DNA damage-regulated autophagy modulator previously implicated in p53-mediated cell death [132]. During *Mm* infection in zebrafish, the induction of *dram1* gene expression is directly linked with innate immunity, as it is independent of p53 and partially dependent on Myd88. Infection of human macrophages further placed NFκB upstream of *DRAM1* gene expression and demonstrated colocalization of DRAM1 protein with *Mtb* [57]. Deficiency in either Myd88 or Dram1 reduces GFP-Lc3 recruitment to *Mm* in zebrafish and impairs the ability to contain *Mm* inside macrophages. Overexpression of Dram1 has the opposite effect, promoting the intracellular killing of *Mm* in zebrafish through enhanced autophagosome formation and autophagic flux [57]. This Dram1-mediated enhancement of autophagy requires the function of the ubiquitin receptor p62 and the stimulator of interferon genes, Sting (Tmem173), previously implicated in the ESX-1-dependent autophagic response to *Mtb* [124]. *DRAM1* induction is associated with the type I interferon-responsive gene signature of human patients with active TB [57, 93]. Recent work shows that cyclic GMP-AMP synthase (cGAS) forms the mechanistic link between the production of type I interferons and the activation of autophagy [133–135]. cGAS functions as a cytosolic sensor of *Mtb* DNA, resulting in the production of cGAMP as a second messenger that activates STING and interferon production [133–135]. cGAS is also required for autophagic targeting of *Mtb* and cGAS-deficient mice are more susceptible to *Mtb* infection [133, 135]. Therefore, despite that the type I interferon response is generally associated with inflammation and disease progression, the same mechanism that triggers this response also activates essential antibacterial functions. The mechanism by which DRAM1 may stimulate the cGAS-dependent autophagic targeting of mycobacteria will require further studies that hopefully will also provide new clues for host-directed anti-TB therapy.

## Concluding remarks

The main strength of the zebrafish model for TB research is the optical access in embryos and larvae to the early stages of granulomas that develop in the context of innate immunity. The ease of genetic and pharmacological manipulation in embryos and larvae has helped to gain better understanding of the roles of macrophages and neutrophils in early pathogenesis and has revealed molecular mechanisms that are exploited by virulent mycobacteria to promote their expansion and dissemination inside the infected host. It can be expected that zebrafish embryos and larvae will also prove useful for in vivo investigation of the epigenetic mechanisms underlying trained innate immunity, which has recently emerged as a new concept in immunology [136]. Trained innate immunity has been implicated in non-specific protective effects of the BCG vaccine against non-mycobacterial diseases and cancers and could also play an important role in tuberculosis [137]. Autophagy is another new immunological paradigm, critical for defense against mycobacteria and also linked with trained innate immunity [119, 138]. In vivo visualization of the autophagic response to mycobacteria in zebrafish larvae has supported many previous in vitro studies that pointed to the central role of autophagy in host defense [57, 86]. Despite limitations in immunological reagents and characterization of the adaptive immune system, adult zebrafish also are a useful addition to TB research, in particular because of the similarities in the structure of fish and human TB granulomas [14, 15, 35]. Zebrafish models are now increasingly applied for translational research into host-directed therapies for tuberculosis [5, 9, 10, 54, 139]. Potential drug targets emerging from this work include pathways involved in macrophage migration, inflammation, cell death regulation, hypoxia signaling, angiogenesis, and mTOR-independent autophagy [31, 55–59, 91, 112, 122]. Targeting the host avoids direct selective pressure on bacteria and therefore has lower risk of drug resistance development [5]. However, possible side effects on the host are a major concern. In view of the many useful characteristics discussed above, zebrafish can play an important role in assessing developmental toxicity and characterizing the mechanisms of drug action in vivo.
